# Piezoelectric Transducer-Based Structural Health Monitoring

**DOI:** 10.3390/s24113438

**Published:** 2024-05-27

**Authors:** Lingyu Yu, Yanfeng Shen, Victor Giurgiutiu

**Affiliations:** 1 Department of Mechanical Engineering, University of South Carolina, 300 Main Street, Columbia, SC 29208, USA; victorg@sc.edu; 2 University of Michigan–Shanghai Jiao Tong University Joint Institute, Shanghai Jiao Tong University, Shanghai 200240, China; yanfeng.shen@sjtu.edu.cn

Piezoelectric effects were first discovered more than a hundred years ago and, since then, have been widely used across various fields. Piezoelectric transducers are mostly made with piezoceramics and piezopolymers. The recent two decades have witnessed tremendous advancements and interesting applications of these types of transducers, including their applications for damage detection and structural health monitoring. This Special Issue is dedicated to all types of piezoelectric transducers that are designed or applied for structural health monitoring (SHM). This Special Issue contains eight full articles and two letters.

The eight full articles in this Special Issue represent exciting research work in different aspects of piezoelectric transducers in SHM. Several papers are tackling damage detection and localization with imaging-based methods. The contribution 1 showed how piezoelectric wafer sensors were used for Lamb wave excitation and sensing in a composite plate for defect detection with a combined wavelet transform and convolution-based Neural Network method. Contribution 2 presented a Time Reversal-based Multiple Signal Classification (TR-MUSIC) algorithm via Lamb wave generation and reception with a piezoelectric array to detect multiple defects in a metallic plate structure. Contribution 3 demonstrated how piezoelectric transducers could be combined with a noncontact laser Doppler vibrometer to obtain Lamb wave wavefield data and detect fatigue cracks with a novel cross-correlation imaging algorithm. Some papers also discussed different applications of piezoelectric transducers. Contribution 4 investigated the near-surface mounted (NSM) fiber-reinforced polymer (FRP) strengthened concrete beam with electromechanical impedance obtained using piezoelectric patch transducers with concurrent influence from mechanical and thermal loadings; contribution 5 tackled underwater cracks in concrete structures based on a cement-based piezoelectric intelligent module array for localization and quantification of the cracks. Contribution 6 applied piezoelectric transducers on a lug shaft and analyzed electromechanical impedance to detect cracks within it. Application development is also included in this Special Issue. Contribution 7 addressed the development of a large-scale piezoelectric network layer combined with a network splitting recombination-based integration strategy and studied system performance with possible effects from various operation conditions. Contribution 8, on the other hand, showcased the development of an embedded piezoelectric diagnostic layer into thick composite materials and compared it to commonly used surface-mounted applications.

The two letters in this Special Issue cover some brief descriptions of recent results. Contribution 9 elucidated how piezoelectric waveguide transducers were developed toward the online monitoring of high-temperature critical components through the adoption of brazing filler metals. Contribution 10 demonstrated a closed-form method for acoustic emission source localizations using complete time difference of the arrival method when acoustic velocity is unknown.

The Guest Editors would like to thank all authors contributing to this Special Issue and for bringing frontline research to the journal of *Sensors*. We would also like to thank the expert reviewers who provided insights in support of the high-quality articles in this field of study. 

